# Comprehensive Clinical and Neuropathological Analysis of Down Syndrome Over Seven Decades of Life

**DOI:** 10.1002/alz.092874

**Published:** 2025-01-03

**Authors:** Sonal Sukreet, Vanessa Goodwill, Jennifer Ngolab, H Y. Kim, Solana Leisher, Sahar N Salehi, Michael Rafii, Annie Hiniker, Robert A. Rissman

**Affiliations:** ^1^ University of California, San Diego, La Jolla, CA USA; ^2^ University of California San Diego, San Diego, CA USA; ^3^ University of Southern California, San Diego, CA USA; ^4^ University of California‐San Diego, La Jolla, CA USA; ^5^ UC San Diego, LA Jolla, CA USA; ^6^ Alzheimer’s Therapeutic Research Institute, San Diego, CA USA; ^7^ University of Southern California, Los Angeles, CA USA; ^8^ Alzheimer’s Therapeutic Research Institute, Keck School of Medicine, University of Southern California, San Diego, CA USA; ^9^ Department of Neurosciences, University of California San Diego, La Jolla, CA USA

## Abstract

**Background:**

Individuals with Down Syndrome (DS) exhibit a unique aging profile and have early‐onset Alzheimer’s Disease (AD) due to a triplication of the amyloid precursor protein (APP) gene on chromosome 21.

**Method:**

Here, we present a study of a 73‐year‐old non‐Hispanic White female with DS who underwent extensive clinical assessments for several decades. She had a cognitive and functional impairment consistent with DS and AD‐like symptoms. Post‐autopsy we did neuropathological and immunohistochemical analysis of the brain and retina.

**Result:**

Neuropathological and immunohistochemical analysis of the brain and retina revealed age and disease‐related changes. Numerous senile plaques, neurofibrillary tangles, Lewy bodies, and cerebral amyloid angiopathy were found throughout cortical and subcortical regions of the brain. Additionally, aberrant TAR DNA‐protein (TDP) 43 accumulation was observed in the amygdala and hippocampus. Immunofluorescent analysis of retinal tissue revealed the presence of amyloid deposits and phosphorylated tau in the ganglion cell and inner plexiform layer of the retina. Whole‐genome sequencing identified two SNPs, rs2802292 and rs4977756 (p < 0.05), linked to longer lifespans.

**Conclusion:**

This case report provides a comprehensive look into the clinical and neuropathological correlates of DS and documents unique clinical and pathological changes over 7 decades of life. We also explored hypotheses and potential mechanisms underlying increased lifespan in certain DS individuals despite progressive intellectual impairment and neurodegenerative pathologies.

